# The Impact of Tooth Retention on Health and Quality of Life in Older Adults

**DOI:** 10.1093/geroni/igab046.2334

**Published:** 2021-12-17

**Authors:** Adejare (Jay) Atanda, Alicia Livinski, Darien Weatherspoon, Paul Fontelo, Shahdokht Boroumand

**Affiliations:** 1 National Institutes of Health, Baltimore, Maryland, United States; 2 NIH, Bethesda, Maryland, United States

## Abstract

America is aging rapidly, and older adults (age ≥65 y) are retaining more of their natural teeth, a trend expected to continue. Although much is known about the impact of complete tooth loss on overall health and well-being, less is known about the effect of partial tooth loss. We conducted a systematic review to advance our understanding of the impact of retaining ≥20 teeth on health and quality of life (QoL) in older adults using two tooth retention concepts – shortened dental arch (SDA) and functional dentition (FD). We searched seven scientific databases from 1981–2019 for publications on tooth retention and outcomes and impact on health and QoL. Ninety-six studies were included in this review. Most were assessed with low risk of bias (n=74) and of good quality (n=73) using the revised Cochrane Risk of Bias tool and Newcastle-Ottawa Scale. Tooth retention was defined as FD in 82 studies, SDA in 10 studies, and four studies used both. Most were cross-sectional and only seven were from the US. We found an increasing trend among published studies in using FD and SDA to describe natural dentition retention (50 articles in 2015-19 vs one in 1995-99). In general, having <20 teeth was associated with increased likelihood for functional dependence, onset of disability, declines in higher-level functioning, and lower QoL. New information is needed to facilitate clinical decision-making, care-giving, and to help health providers better meet the future oral health needs of an aging US population.

